# Comparison of anxiety and depression status between office and manufacturing job employees in a large manufacturing company: a cross sectional study

**DOI:** 10.1186/s40557-016-0134-z

**Published:** 2016-09-15

**Authors:** WonYang Kang, Won-Ju Park, Keun-Ho Jang, Hyeong-Min Lim, Ji-Sung Ann, Seung-hyeon Cho, Jai-Dong Moon

**Affiliations:** 1 Department of Occupational and Environmental Medicine, Chonnam National University Hwasun Hospital, 160, Ilsim-ri, Hwasun-eup, Hwasun-gun, Jeollanam-do 58128 Republic of Korea; 2 Department of Occupational and Environmental Medicine, Mokpo Christian Hospital, Mokpo, Jeollanam-do Republic of Korea

**Keywords:** Anxiety, Depression, Office job, Manufacturing job, Working hours

## Abstract

**Background:**

The aim of this study was to investigate whether type of work is associated with anxiety and depression using the Hospital Anxiety and Depression Scale (HADS). Additionally, we investigated the impact of number of working hours on anxiety and depression.

**Methods:**

A total of 1774 workers participated and completed the HADS to determine their levels of anxiety and depression. All subjects were employed at one of two manufacturing plants for the same company. Of all participants, 222 were employed in office jobs and 1552 in manufacturing jobs.

**Results:**

Results of multivariate logistic regression analysis including age, sex, body mass index, smoking status, alcohol consumption, regular exercise, factory region, and working hours, indicated that employment in an office job was associated with a 2.17-fold increase in the odds of anxiety compared to a manufacturing job (odds ratio [OR] = 2.17; 95 % confidence interval [CI], 1.24–3.80). Office jobs were also associated with a 1.94-fold increase in the odds of depression (OR = 1.94; 95 % CI, 1.34–2.82). In addition, number of hours worked was significantly associated with depression, and working hours significantly modified the effect of office job employment on the risk of depression.

**Conclusions:**

Office job workers had higher levels of anxiety and depression than those working in manufacturing jobs. Our findings suggest that occupational physicians should consider the organizational risks faced by office job employees, and consider the differences in psychological health between office and manufacturing job workers when implementing interventions.

## Background

Anxiety and depression are the most common psychological health problems. In the prevalence studies of anxiety disorders published between 1980 and 2009, the global prevalence of anxiety disorder was 7.3 % (4.8–10.9 %). According to the sixth Korean National Health and Nutrition Examination Survey in 2014, the prevalence rate of depression was 6.6 % [1, 2]. Furthermore, a national survey in South Korea in 2005 estimated that the total cost of depression was $ 4,049 million [3].

Not only are there negative mental health effects for general population, but there are also deleterious effects on mental health for employees, with an estimated prevalence of about 10 to 20 % worldwide [4, 5]. Mental health problems in the workplace may lead to economic burden, as well as increased absenteeism, labor compensation claims, high medical cost, and reduced productivity [6]. Therefore, workplace psychological health interventions are required. Moreover, this scope must be expanded beyond individual factors to focus on organizational factors in the workplace.

Organizational factors that may impact mental health in the workplace are heterogeneous according to work type or occupation classification [7, 8]. Additionally, work characteristics and labor environment, which can reveal the status of mental health, differ across occupational groups. However, there are relatively few studies that demonstrate differences in mental health status based on different types of work. The purpose of comparing common mental disorders between work types is to identify organizations with hazardous psychological environments and provide occupational health interventions for vulnerable groups.

In addition to type of work, working hours are a known occupational risk factor for mental health problems. In a systematic review of epidemiological research, long working hours that are greater than 40 h per week or 8 h per day were associated with both depressive state and anxiety [9]. Thus, type of work and working hours may be interrelated in their effect on mental health and both factors should be considered simultaneously. Previous studies have independently investigated the relationship between occupational group and working hours on mental health. In this study, we aimed to evaluate the association between type of work and common mental disorders (anxiety and depression) by utilizing the Hospital Anxiety and Depression Scale (HADS). Additionally, we analyzed how working hours interact as part of this relationship.

## Methods

### Participants

We conducted a cross-sectional study that included subjects from two large-scale manufacturing factories (Factory A and Factory B) that were part of the same company. The 3721 full-time employees at the factories underwent periodic health examinations between October 2015 and November 2015. The researchers requested that all employees complete a questionnaire consisting of questions regarding mental health as measured by the HADS. Of these employees, 1790 individuals (996 individuals = Factory A; 794 individuals = Factory B) voluntarily completed the questionnaire (response rate = 47.7 %). After excluding 16 individuals who suffered from severe diseases (e.g., cardiovascular or cerebrovascular disease), the total sample included 1774 participants. Study participants underwent blood analysis and clinical examinations to evaluate anxiety and depression risk factors and behavioral factors. Each subject provided written informed consent prior to participation. Physicians explained the purpose, methods, and precautions of this study. Tests were conducted only after informed consent was obtained from each participant. This study was approved by the Chonnam National University Hwasun Hospital Institutional Review Board (CNUHH-2016-072).

### Study variables

The two factories provided data that included information about individual departments and the associated primary tasks. The Korean Standard Classification of Occupations (KSCO) includes the following ten major occupation categories: managers; professionals; clerks; service workers; sales workers; skilled agricultural, forestry and fishery workers; craft and related trades workers; machine operating and assembling workers; elementary workers; and armed forces [10]. In the current study, the manufacturing companies included managers, professionals, clerks, and craft and related trades workers. Subsequently, we classified participants into an office job group and a manufacturing job group based on the main task performed by the participant. Specifically, categorizations into office or manufacturing job groups were based on whether the different occupational classes included non-manual labor or manual labor, which are analogous to white- and blue-collar positions, respectively. Managers, some professionals, and clerks were allocated to office jobs in the manufacturing company. Other professionals as well as craft and related trades workers were allocated to manufacturing jobs. For example, administration-related clerks were classified as holding office jobs, while rolling mill operators were classified as having a manufacturing job.

All participants were interviewed by a clinician during their periodic health examination. To determine worked hours, subjects were asked about the average number of hours they worked per week. This resulted in categorization into four groups: 40 h or less (reference category), 41–51 h, 52–59 h, and 60 h and over [9]. Since they were likely confounders, age, sex, body mass index (BMI), smoking status, drinking status, exercise, and factory region were included in the model [11, 12]. Age was divided into four groups (20–29, 30–39, 40–49, and 50–59).

Height and weight were measured while the subjects were barefoot. BMI was calculated using the formula: weight (kg)/height^2^ (m^2^). Smoking status was categorized into never, past, and current. According to the National Institute on Alcohol Abuse and Alcoholism, one standard drink contains roughly 14 g of pure alcohol, which amounts to one can of beer (330 mL), one glass of wine (150 mL), or one shot of hard liquor (40 mL) [13]. Subjects who had a mean daily alcohol consumption of more than a standard drink for the past month were classified as drinkers, while the rest were classified as non-drinkers. Subjects who rigorously exercised for 30 min or longer (to the point that they sweated) at least five times a week were assigned to the physical activity group [14]. The company assessed consists of two main factories, one of which is located in a city and the other in a rural area.

### Outcome variables

In this study, the dependent variable was anxiety and depression, which was evaluated using the HADS. Participants independently completed the HADS prior to the clinical exam. The HADS is a self-assessment scale consisting of 14 items that are scored on a 4-point scale that measures the presence and severity of anxiety and depression separately. A score greater than or equal to 8 is regarded as the clinical cut-off [15]. The HADS has demonstrated acceptable reliability and validity in a general population [16]. In addition, this tool has been found to be adequate for detecting anxiety and depression symptoms in a workplace population [17]. In this study, Cronbach’s α scores for anxiety and depression were 0.85 and 0.78, respectively.

### Statistical analyses

The subjects were divided in manufacturing and office job groups. A chi-square test was used to compare and analyze data with respect to age, sex, BMI, smoking status, alcohol consumption, regular exercise, factory region, working hours, and anxiety and depression. The associations between type of work and HADS scores for anxiety and depression were analyzed by performing multivariate logistic regression analyses with two different models. The models were as follows: (1) unadjusted model; and (2) adjusted model for age, sex, BMI, smoking status, alcohol consumption, regular exercise, factory region, and working hours. To understand the impact of working hours on anxiety and depression as an effect modifier, we also used a multivariate logistic regression analysis based on the type of work by stratification of working hours and adjustment of covariates. The interaction between the type of work and working hours was also analyzed. Results were expressed as odds ratios (OR) with 95 % confidence intervals (CI). Statistical significance was set at *p* < 0.05. All statistical tests were performed using SPSS version 18.0 software (SPSS, Inc. Chicago, IL, USA).

## Results

Table 1 summarizes the subjects’ characteristics. A total of 1552 (87.5 %) of subjects performed manufacturing jobs and 222 (12.5 %) were classified as performing office jobs. Subjects ranged in age from 20 to 59 years. With the exception of BMI and drinking status, there were statistically significant differences in the distribution of all other general characteristics between the manufacturing and office job groups. Additionally, both anxiety and depression symptoms were more prevalent among office than manufacturing workers.

**Table 1:** General characteristics of subjects

	Manufacturing job (*N* = 1552)	Office job (*N* = 222)	
	N (%)	N (%)	*p* value^*^
Age (years)			<0.01
20–29	47 (3.0)	44 (19.8)	
30–39	156 (10.1)	110 (49.5)	
40–49	847 (54.6)	52 (23.4)	
50–59	502 (32.3)	16 (7.2)	
Sex			<0.01
Men	1521 (98.0)	190 (85.6)	
Women	31 (2.0)	32 (14.4)	
BMI (kg/m^2^)			0.21
< 25	839 (54.1)	110 (49.5)	
≧25	713 (45.9)	112 (50.5)	
Smoking			<0.01
Never	423 (27.3)	99 (44.6)	
Past	502 (32.3)	46 (20.7)	
Current	627 (40.4)	77 (34.7)	
Drinking			0.90
Non-drinker	426 (27.4)	60 (27.0)	
Drinker	1126 (72.6)	162 (73.0)	
Exercise			<0.01
Physical activity	147 (9.5)	8 (3.6)	
Physical inactivity	1405 (90.5)	214 (96.4)	
Factory region			<0.01
Factory A	844 (54.4)	144 (64.9)	
Factory B	708 (45.6)	78 (35.1)	
Working hours (weekly)			<0.01
≦40	315 (20.3)	21 (9.5)	
41–51	600 (38.7)	71 (32.0)	
52–59	519 (33.4)	66 (29.7)	
≧60	118 (7.6)	64 (28.8)	
Anxiety			<0.01
No	1447 (93.2)	190 (85.6)	
Yes	105 (6.8)	32 (14.4)	
Depression			<0.01
No	1146 (73.8)	137 (61.7)	
Yes	406 (26.2)	85 (38.3)	

*BMI* body mass index

^*^ *P* value was calculated by Chi-square test

Table 2 presents the crude and adjusted models for the association between each variable and anxiety. Working an office job was significantly associated with increases in the odds of having anxiety compared with manufacturing job in both the crude model (OR = 2.32; 95 % CI, 1.52–3.55) and adjusted model (OR = 2.17; 95 % CI, 1.24–3.80). The odds of having anxiety increased as working hours increased. After adjusting all covariates, the association between working hours and anxiety decreased slightly.

**Table 2:** The association between type of work and anxiety

	Crude OR (95 % CI)	Adjusted^a^ OR (95 % CI)
Age (years)		
20–29	1.00	1.00
30–39	0.90 (0.40 to 2.02)	0.87 (0.37 to 2.01)
40–49	0.68 (0.32 to 1.41)	0.91 (0.40 to 2.09)
50–59	0.80 (0.38 to 1.71)	1.21 (0.51 to 2.85)
Sex		
Men	1.00	1.00
Women	2.06 (0.99 to 4.27)	2.63 (1.10 to 6.31)
BMI (kg/m^2^)		
< 25	1.00	1.00
≧25	1.26 (0.89 to 1.79)	1.29 (0.91 to 1.85)
Smoking		
Never	1.00	1.00
Past	1.32 (0.83 to 2.08)	1.58 (0.94 to 2.66)
Current	1.26 (0.81 to 1.97)	1.59 (0.96 to 2.63)
Drinking		
Non-drinker	1.00	1.00
Drinker	1.32 (0.87 to 1.99)	1.28 (0.82 to 1.99)
Exercise		
Physical activity	1.00	1.00
Physical inactivity	1.85 (0.85 to 4.02)	1.51 (0.69 to 3.33)
Factory region		
Factory A	1.00	1.00
Factory B	1.39 (0.98 to 1.97)	1.19 (0.80 to 1.77)
Working hours (weekly)		
≦40	1.00	1.00
41–51	1.61 (0.89 to 2.93)	1.42 (0.77 to 2.65)
52–59	2.13 (1.18 to 3.85)	1.85 (0.97 to 3.52)
≧60	2.94 (1.49 to 5.83)	2.15 (0.99 to 4.64)
Type of work		
Manufacturing job	1.00	1.00
Office job	2.32 (1.52 to 3.55)	2.17 (1.24 to 3.80)

*BMI* body mass index

^a^Adjusted for age, sex, BMI, smoking, drinking, exercise, factory region, working hours and type of work

Table 3 presents the associations between each variable and depression. Results revealed that the odds of having depression also increased in the office job group compared to manufacturing job group (OR = 1.75; 95 % CI, 1.31–2.35). After adjusting all covariates, the association between working and office job and depression was strengthened (OR = 1.94; 95 % CI, 1.34–2.82). Additionally, as subjects’ working hours increased, the likelihood of depression increased. Moreover, those who worked 60 h or over per week had a statistically significant increase in the odds of having depression compared to those who worked 40 h or less in both the crude model (OR = 2.38; 95 % CI, 1.60–3.54) and adjusted model (OR = 1.87; 95 % CI, 1.19–2.94).

**Table 3:** The association between type of work and depression

	Crude OR (95 % CI)	Adjusted^a^ OR (95 % CI)
Age (years)		
20–29	1.00	1.00
30–39	1.74 (0.98 to 3.11)	1.53 (0.84 to 2.80)
40–49	1.70 (0.99 to 2.90)	1.95 (1.10 to 3.50)
50–59	1.33 (0.77 to 2.31)	1.67 (0.91 to 3.05)
Sex		
Men	1.00	1.00
Women	1.13 (0.70 to 1.96)	1.55 (0.83 to 2.89)
BMI (kg/m^2^)		
< 25	1.00	1.00
≧25	1.14 (0.93 to 1.41)	1.14 (0.92 to 1.41)
Smoking		
Never	1.00	1.00
Past	1.45 (1.10 to 1.93)	1.58 (1.17 to 2.15)
Current	1.84 (1.41 to 2.40)	2.10 (1.57 to 2.82)
Drinking		
Non-drinker	1.00	1.00
Drinker	1.02 (0.81 to 1.30)	0.88 (0.69 to 1.13)
Exercise		
Physical activity	1.00	1.00
Physical inactivity	1.91 (1.24 to 2.92)	1.65 (1.07 to 2.56)
Factory region		
Factory A	1.00	1.00
Factory B	1.37 (1.11 to 1.68)	1.16 (0.91 to 1.47)
Working hours (weekly)		
≦40	1.00	1.00
41–51	1.29 (0.94 to 1.77)	1.19 (0.85 to 1.66)
52–59	1.70 (1.24 to 2.34)	1.50 (1.05 to 2.13)
≧60	2.38 (1.60 to 3.54)	1.87 (1.19 to 2.94)
Type of work		
Manufacturing job	1.00	1.00
Office job	1.75 (1.31 to 2.35)	1.94 (1.34 to 2.82)

*BMI* body mass index

^a^Adjusted for age, sex, BMI, smoking, drinking, exercise, factory region, working hours and type of work

Table 4 presents the results of the subgroup analyses. Here, it is shown that working hours significantly modified the effects of working an office job for risk of depression symptoms. Specifically, office job workers had a higher risk of experiencing depression symptoms according to the magnitude of the effect, which was increased by working a higher number of hours. In stratified analyses, working an office job for 60 h a week or over was significantly associated with increases in the odds of workers having both anxiety and depression compared to working a manufacturing job.

**Table 4:** Odds ratio of office job for anxiety and depression, stratified according to working hours^a^

	Anxiety		Depression	
	Adjusted^b^ OR (95 % CI)	*p* value of effect modification	Adjusted^b^ OR (95 % CI)	*p* value of effect modification
Working hours (weekly)		0.16		0.01
≦40	3.55 (0.65 to 19.34)		0.72 (0.20 to 2.64)	
41–51	1.38 (0.47 to 4.10)		1.84 (0.93 to 3.65)	
52–59	1.25 (0.42 to 3.77)		1.85 (0.91 to 3.77)	
≧60	5.00 (1.39 to 18.00)		2.62 (1.05 to 6.51)	

^a^Reference group was manufacturing job

^b^Adjusted for age, sex, BMI, smoking, drinking, exercise and factory region

## Discussion

Our results suggest that, as evaluated with the HADS, working an office job elevated the risk of anxiety and depression compared to working a manufacturing job. These effects were more predominant in workers who worked long hours (60 or more hours per week). Our results are consistent with the results of a previous study that compared the prevalence of common mental disorders between occupational groups. Specifically, a national UK survey by Stansfeld et al. showed that plant workers and machine operators (OR = 0.43; 95 % CI, 0.28–0.68) had a reduced risk of common mental disorder compared to managers and administrators [4]. Thus, our study is analogous to their finding that working an office job is a risk factor for common mental disorders. In contrast, in another study, Kim et al. found that blue-collar workers (β: 0.387, *p* = 0.010) had higher depression scores than white-collar workers [18]. The participants in these two studies represented the general population, which included many kinds of businesses. However, different types of businesses have different work characteristics and require different types of labor, even if workers are classified in the same occupational groups. Consequently, previous studies that focus on the general population demonstrate heterogeneous results and are limited in terms of their practical application in the workplace.

In the current study, the risk of anxiety and depression increased as the number of hours worked per week increased. Consistently, when Virtanen et al. conducted a prospective study of 2960 full-time employees they reported that number of working hours was a risk factor for the development of anxiety and depression. Specifically, using a cox proportional hazard model, it was found that the hazard ratio for anxiety symptoms was 1.74 (95 % CI, 1.15–2.61) and 1.66 (95 % CI, 1.06–2.61) for depression symptoms among employees that worked more than 55 h per week compared with employees working less than 40 h per week [19]. Additionally, a study of manufacturing workers conducted by Kato et al. reported that the OR for depressive disorder was 4.5 (95 % CI, 1.80–11.10) times higher for employees working more than 60 h per week than for those working less than 50 h per week [20]. Although there are slight differences between these studies, our study results are consistent with their findings.

In the present study, type of work and working hours were shown to be the workplace risk factors for anxiety and depression. However, little is known about the interaction between type of work and working hours, which have an impact on anxiety and depression. Our study demonstrated that office job workers were more vulnerable to anxiety and depression symptoms than manufacturing job workers, especially among those who worked 60 or more hours per week. In addition, working hours could be an effect modifier between type of work and depression.

Office jobs could increase the risk of anxiety and depression as a result of differences in organizational factors between this job type and manufacturing jobs. Specifically, organizational injustice between the two types of work may play a role in the higher prevalence of anxiety and depression for a number of reasons [21]. First, there was an unequal ratio of input to output between the two types of work. Office workers were likely to receive lower wages for working the same number of hours as manufacturing workers. For example, overtime pay, which was about 13,000 won per hour, was provided under the policy of this company. Overtime pay rule was well applied to manufacturing workers, but not to office workers for several reasons. Also, this may be an additional explanation for working hours to act as an effect modifier. Second, office workers’ departments were more likely to be undemocratic and exhibit inconsistent application of policies, procedure, and practices. For instance, a study by Tak reported that conflict between departments and decision-making were the most important factors associated with job stress among office workers [22]. Third, office workers are more likely to have highly demanding jobs with little support and few resources. Subsequently, decreases in control, autonomy, and support are potential risk factors for mental health problems [23]. Finally, a major organizational factor that differs between office and manufacturing jobs is related to differences in rates of labor union affiliations. Specifically, manufacturing job workers are reported to be much more affiliated with labor unions than office job workers [24]. At the macro level, union density may be an important factor for explaining workers’ self-reported health and work stress, which could be explained by labor protection [25]. Additionally, office workers are more vulnerable to business restructuring and organizational downsizing, resulting in a lack of long-term job security.

Contrary to the results of the current study, some research has shown that blue-collar work rather than white-collar work is a risk factor of anxiety and depression [18, 26]. Since high educational status and non-manual work were favored in South Korea during the industrialization period, there was discrimination toward individuals employed in blue-collar positions. However, after industrialization, the inclination to favor white-collar work has changed and discrimination has declined. In the past, blue-collar work was associated with the burden of diminished physical health resulting from heavy and prolonged manual labor. That said, working conditions for these skilled occupations might have recently improved. Thus, we assume that social changes and changes in the workplace environment may play a role in creating different results between studies.

The association between working hours and anxiety was weakened after adjusting covariates, and the interaction between working hours and type of work was non-significant. According to our results, we can assume that working hours might have more of an impact on depression than anxiety symptoms. Specifically, office workers were vulnerable to depression symptoms because of organizational injustice, high job demands, and a low level of labor protection. Additionally, when office workers must work long hours, they cannot recover from occupation-related psychosocial strains leading to an inability to escape this vicious cycle. For instance, working long hours leads to less sleep and time to recover from occupational stress and likely reduced workers’ time for leisure-related activities [27]. Hence, working hours could be an effect modifier of pathways from working an office job to depression in the workplace.

The limitations of this study should be considered. First, because the study design was cross-sectional, it was not possible to determine if there was a precise causal relationship. Thus, because a longitudinal design was not utilized, we could not trace workers’ tasks over time or evaluate all workers’ tasks based on type of work. However, job transfers between office and manufacturing jobs rarely occurred in the type of large manufacturing company assessed in this study. Second, we did not consider psychosocial factors such as socioeconomic status, education, and marital status. In this study, income was not used to represent socioeconomic status which could be a confounder of association between type of work and mental health. According to the material of company, there was a gap between office workers and manufacturing workers in the average of total annual income including regular pay, holiday pay, overtime pay and premium pay (office workers : 60 million won, manufacturing workers : 65 million won). We could not get the information about total annual income of study participants individually. If total income was adjusted in this study model, the association between type of work and mental health would be weakened. Additionally, low education level is a known risk factor for mental disorders and, generally, workers in manufacturing jobs had a lower education level than those in office jobs. Considering the high prevalence of anxiety and depression among office job workers, it appears that the impact of education was not stronger than type of work, and there is a possibility that the impact of office jobs on mental health was underestimated in this study. Finally, since this study focused on subjects working in a large manufacturing company representing a single workplace, caution must be used when generalizing these results to other workplaces.

## Conclusion

Our study demonstrated that type of work was associated with increasing the risk of anxiety and depression in workplace. In addition, it was revealed that working hours could be a risk factor for depression and that office jobs and working hours have a combined effect on depression. Although the precise mechanism by which office jobs affect anxiety and depression has not been established, organizational injustice and inequality between occupational classifications might play a role in increasing mental disorders. Thus, our results may have implications for prevention programs in occupational health. In the past, the management of occupational mental health has focused on those engaged in manufacturing work or blue-collar workers. However, it will be important for occupational physicians to identify organizational factors that impact mental health across occupational classifications and administer the most appropriate intervention based on the specific type of work that the employee is engaged in.

## Abbreviations

BMI: Body mass index
CI: Confidence interval
HADS: Hospital anxiety and depression scale
KSCO: Korean standard classification of occupations
OR: Odds ratio
